# Effects of Hormones and Endocrine Disorders on Hair Growth

**DOI:** 10.7759/cureus.32726

**Published:** 2022-12-20

**Authors:** Rishi Hasan, Husain Juma, Fatema A Eid, Hawra A Alaswad, Walaa M Ali, Fatima J Aladraj

**Affiliations:** 1 Dermatology, Global Dermatology, Manama, BHR; 2 General Practice, Taj Medical Center, Budaiya, BHR; 3 General Practice, Ministry of Health, Sanabis, BHR; 4 General Practice, Symmetria Middle East, Tubli, BHR

**Keywords:** hypertrichosis, hyperandrogenism, hormones, hirsutism, endocrine disorders

## Abstract

Hormones have a close association with hair growth; thus, they have a big impact on the hair cycle and hair follicle structure. Many hormones control hair growth, cycle, and density. Hair abnormalities are frequent in therapeutic practice, and they can cause severe emotional discomfort depending on societal and ethnic standards. As a result, disorders that impact the endocrine system can induce a variety of physiological hair growth and cycling alterations. Hirsutism and patterned hair loss have a significant impact on human personality. These illnesses necessitate a comprehensive approach to diagnosis and treatment. The hormonal impact on hair growth and the association of different endocrine disorders with hair changes are briefly discussed here.

## Introduction and background

Hair is a key physical structure that has a significant impact on a person's psychosocial personality. Hair follicles (HFs) are made up of dermal papilla cells (DPCs) formed from mesenchymal cells and epithelial-derived root sheath cells, with mesenchymal-derived DPCs interacting with epithelial-derived root sheath cells [1]. The hair growth cycle consists of three phases. These phases are anagen, catagen, and telogen representing growth, regression, and rest respectively [2]. DPCs also produce and release a number of cytokines that control hair growth and cycle [3].

Hair growth, cycling, and density can all be affected by endocrine disorders, and a thorough examination may discover the underlying problem. Hormonal abnormalities include hypertrichosis, hirsutism, and alopecia areata. However, the task is complicated by a lack of data and a discrepancy in the literature on the effect of hormonal influence on the hair cycle, which has not been properly examined. This article briefly discusses the hormonal impact on hair growth and the association of different endocrine disorders with hair changes.

## Review

Hormones and their effects on hair

Androgens

They are the primary regulators of proper hair growth in humans [4]. They operate on hair follicles through interaction with intracellular receptors inside DPCs, depending on where the hair is located over the body [5,6]. Androgen's major effect is to interact with androgen receptors in DPCs [7]. The impact of androgens is to change the vellus hairs which are thin, short, and straight into terminal hairs which are darker, bigger, and curlier in sex-specific parts of the body [8]. In both sexes, androgens convert axillary and groin vellus hair follicles, as well as the vellus hair in the trunk and beard area of males, into terminal hair during puberty [9]. The pathophysiology and course of patterned hair loss are influenced by circulating androgens. This is based on the observation that unless testosterone is provided, eunuchs and castrated boys do not acquire male-patterned hair loss [10]. On DPCs, when androgen receptors are occupied by di-hydro testosterone and testosterone, they mediate alteration in the production of soluble factors, influencing the activity and maturity of variable cells, in particular, hair follicle keratinocytes, which results in male patterned hair loss. As a result, scalp hairs get increasingly thinner [11].

Oestrogen

Androgens undergo peripheral aromatisation in adipose tissue to make oestrogens. In females, oestrogen is necessary for the development of pubic and axillary hair. Oestrogens, on the other hand, have long been known to have a significant impact on hair follicle changes affecting the growth of hair follicles through binding to high-affinity, locally produced oestrogen receptors [12]. Due to the depletion of finite ovarian follicles, menopause is accompanied by a decrease in oestrogen and progesterone release, which can lead to hair and skin illnesses [13,14]. After menopause, the higher frequency of developing female pattern hair loss indicates that oestrogens play a role in the stimulation of hair growth. In postmenopausal women, the etiologies as well as management strategies of certain conditions including hair loss and hirsutism are quite different compared to premenopausal women. During pregnancy, high circulatory levels of oestrogen may contribute to the prolonging of anagen, while a drop in circulatory levels of oestrogen in the post-partum period is thought to contribute to post-partum hair loss which is known as telogen gravidarum [15]. The fact that sex steroid hormones exhibit an extraordinarily effective inhibitory function on the hair cycle, notably through halting the clock of the hair cycle in telogen, is perhaps the most exciting feature of oestrogen biology from the standpoint of hair study [12,16].

Growth Hormone

This enhances androgen's influence on sexual hair growth. In growth hormone-deficient hypogonadal males, the required level of testosterone to stimulate hair growth in the axilla may reach around 5 times the normal testosterone level in growth hormone-sufficient hypogonadal boys [9].

Insulin and Insulin-Like Growth Factor (IGF)

These hormones play a part in hair development stimulation and work in tandem with androgens. Hyperinsulinemia may boost di-hydro testosterone synthesis by inducing 5α reductase activity.

Prolactin

Prolactin is a hormone that plays a function in lactation, reproduction, angiogenesis, osmoregulation, and hair development [17]. In females, prolactin promotes the hair shaft lengthening in the front-temporal region of the scalp while inducing catagen in male occipital scalp hair follicles [18,19]. Prolactin excess is linked to hirsutism in clinical studies, most likely due to the stimulation of hyperandrogenism.

Melatonin

Melatonin is a hormone that regulates the rhythm of a variety of physiological systems. Melatonin receptors can be present in sweat glands, blood vessels endothelium, epidermal keratinocytes, and dermal fibroblasts, in addition to hair follicle cells. Melatonin affects hair pigmentation primarily by increasing the number of melanocytes, as well as its growth, most likely via accelerating the anagen phase. The ability of melatonin to control the hair follicle response to oestrogens, weakening oestrogen receptors expression in the hair follicle, is one of its most essential functions. One of the roles of melatonin is to activate nuclear factor erythroid-2-related factor 2, which has a significant impact on the protection of hair follicles from oxidative stress and thus to inhibit hair growth suppression [20,21].

Cortisol

Hair loss has become more common in women and young people, and it is thought that stresses, rather than genetic factors, are to blame. As a result, accumulating data on the impact of stress on HFs and their constituent cells is critical for hair loss treatment [22]. The association between stress hormones and hair loss, on the other hand, is poorly understood. Cortisol is recognized to affect the function and cyclic regulation of the hair follicle [23]. High levels of cortisol are linked to a decrease in the formation and early breakdown of hair follicle modulators such as proteoglycans and hyaluronans, which are essential for hair follicle activity [24]. Corticotropin-releasing factor (CRF), in particular, is a key HPA axis hormone in the peripheral stress response. Hair shaft elongation is greatly inhibited by CRF activation [25].

Thyroid

Thyroid receptors were detected on the outer root sheath cells of hair follicles. The thyroid hormone is believed to be responsible for regulating the hair cycle's frequency [26]. Hypothyroidism causes a decrease in anagen frequency, whereas hyperthyroidism causes thin hairs.

Endocrine conditions causing abnormal/excessive hair growth

Hirsutism

Excessive terminal hair in parts of the female body that are androgen-dependent is a symptom of elevated androgen activity in the hair follicle. Worldwide, hirsutism in females frequently leads to psychological suffering, lack of confidence, and cosmetic embarrassment. It is a clinical symptom of hyperandrogenemia (abnormally high levels of androgens detected in the blood) [27]. The prevalence of hirsutism ranges from 4-11 percent, however, Asians appear to have a lower rate [28]. With advancing age, the prevalence and severity of hirsutism decrease (especially in postmenopausal women) [29]. In 75 percent of patients with hyperandrogenemia, hirsutism is present [22]. Polycystic ovarian syndrome is the most frequent cause of hirsutism, accounting for more than 70% of cases [30]. Idiopathic hirsutism affects 5-17 percent of hirsutism sufferers, depending on ethnicity and geographic location. The underlying cause of hirsutism in about 1-8% of women is non-classical congenital adrenal hyperplasia due to a 21-hydroxylase deficiency [31]. The majority of females who experience frontal-central pattern hair loss do not have elevated levels of androgen and do not manifest with hyperandrogenism symptoms, such as hirsutism or irregular periods/anovulation [32].

Hypertrichosis

Hypertrichosis is the presence of abundant body hair that is not caused by androgen. It can be hereditary or acquired. Cushing's disease, acromegaly, and hypothyroidism are the main endocrine causes of acquired hypertrichosis [33]. Physical treatment approaches, which are frequently paired with medical therapy, can result in significant cosmetic improvement.

Endocrine conditions associated with hair loss

In both males and females, patterned hair loss is closely associated with sex hormones level, because it is related to alterations in the androgen receptor and responds to antiandrogen medication [34].

Male Pattern Hair Loss

Male pattern hair loss has been classified as androgen-dependent [34]. A study by Sanke et al., comparing the hormonal profile of early androgenetic alopecia in men with the phenotypic equivalent of polycystic ovarian syndrome in women, on the other hand, have discovered hormonal imbalances in androgenetic alopecia similar to polycystic ovarian syndrome [35]. Androgenetic alopecia, or male pattern hair loss, is a common, age-dependent, highly heritable disorder marked by progressive front-temporal and vertex hair loss [9]. The pattern of inheritance is polygenic [9]. The condition is hormone-related with androgen being a key hormonal influence.

Female Pattern Hair Loss

Female pattern hair loss (FPHL) is defined as the progressive shrinking of hair follicles, which mainly occurs in genetically predisposed females and is rarely associated with endocrine disorders. Hair loss in women can be a severe source of psychological stress and morbidity, which is unsurprising. Female patterned hair loss affects 6 to 64.4 percent of women, with Asians having a somewhat lower incidence [36]. Female pattern hair loss is prevalent in the 3rd and 5th decades of women's life. The prevalence rises with age, starting at 1.3 percent in the 18-29 year age group and rising to 10.3 percent in the seventh decade and 11.8 percent thereafter [37]. Female pattern hair loss is a complex phenomenon that is associated with multiple contributing factors. The condition is more common in genetically predisposed women with abnormal cycling of the hair follicle, which results in the terminal hair follicles being transformed into shorter and finer vellus hair follicles. There is a steady reduction in the size of the dermal papilla under the effect of sex hormones, as well as a reduction in anagen duration and a prolongation of telogen [36]. Diffuse telogen hair loss affects around 50 percent of people with hyperthyroidism and 33 percent of people with hypothyroidism respectively [38]. Severe hair loss does not always reflect a severe endocrine imbalance. Hypothyroidism in its early stages may present with telogen effluvium as the initial presenting sign. On histopathology, a reduction in the terminal/vellus ratio and follicular miniaturization can distinguish early female patterned hair loss from telogen effluvium. Acute telogen effluvium can sometimes reveal latent female patterned hair loss.

## Conclusions

It can therefore be concluded that this piece of work covered various hormones and their effects on hair growth and also touched briefly on the association between various endocrine conditions and their influence on hair changes. The hair cycle and hair follicle structure are greatly impacted by various hormones. Hair changes are prevalent in endocrine illnesses and might be the first indicator of underlying hidden endocrinopathy. Hence, this article is important to reach a comprehensive approach in order to diagnose and treat various hair disorders. Despite the fact that various hormonal mechanisms and pathways influencing hair development have been documented here, more comprehensive evidence and research are needed to understand the whole process of hormonal effect on the hair cycle.

## References

[1] Millar SE (2002). Molecular mechanisms regulating hair follicle development. J Invest Dermatol.

[2] Stenn KS, Paus R (2001). Controls of hair follicle cycling. Physiol Rev.

[3] Yang CC, Cotsarelis G (2010). Review of hair follicle dermal cells. J Dermatol Sci.

[4] Lai JJ, Chang P, Lai KP, Chen L, Chang C (2012). The role of androgen and androgen receptor in skin-related disorders. Arch Dermatol Res.

[5] Mason KA, Schoelwer MJ, Rogol AD (2020). Androgens during infancy, childhood, and adolescence: physiology and use in clinical practice. Endocr Rev.

[6] Chaturvedi AP, Dehm SM (2019). Androgen receptor dependence. Adv Exp Med Biol.

[7] Brown TM, Krishnamurthy K (2020). Histology, hair and follicle. https://www.ncbi.nlm.nih.gov/books/NBK532929/.

[8] Kini S, Ramalingam M (2018). Hirsutism. Obstet Gynaecol Reprod Med.

[9] Rosenfield RL (2005). Hirsutism and the variable response of the pilosebaceous unit to androgen. J Investig Dermatol Symp Proc.

[10] Hamilton JB (1942). Male hormone stimulation is prerequisite and an incitant in common baldness. Am J Anat.

[11] Bahta AW, Farjo N, Farjo B, Philpott MP (2008). Premature senescence of balding dermal papilla cells in vitro is associated with p16(INK4a) expression. J Invest Dermatol.

[12] Ohnemus U, Uenalan M, Conrad F (2005). Hair cycle control by estrogens: catagen induction via estrogen receptor (ER)-alpha is checked by ER beta signaling. Endocrinology.

[13] Bruce D, Rymer J (2009). Symptoms of the menopause. Best Pract Res Clin Obstet Gynaecol.

[14] Al-Azzawi F, Palacios S (2009). Hormonal changes during menopause. Maturitas.

[15] Simpson D, Curran MP, Perry CM (2004). Letrozole: a review of its use in postmenopausal women with breast cancer. Drugs.

[16] Paus R, Foitzik K (2004). In search of the "hair cycle clock": a guided tour. Differentiation.

[17] Castle-Miller J, Bates DO, Tortonese DJ (2017). Mechanisms regulating angiogenesis underlie seasonal control of pituitary function. Proc Natl Acad Sci U S A.

[18] Foitzik K, Krause K, Conrad F, Nakamura M, Funk W, Paus R (2006). Human scalp hair follicles are both a target and a source of prolactin, which serves as an autocrine and/or paracrine promoter of apoptosis-driven hair follicle regression. Am J Pathol.

[19] Langan EA, Ramot Y, Goffin V, Griffiths CE, Foitzik K, Paus R (2010). Mind the (gender) gap: does prolactin exert gender and/or site-specific effects on the human hair follicle?. J Invest Dermatol.

[20] Janjetovic Z, Jarrett SG, Lee EF, Duprey C, Reiter RJ, Slominski AT (2017). Melatonin and its metabolites protect human melanocytes against UVB-induced damage: involvement of NRF2-mediated pathways. Sci Rep.

[21] Haslam IS, Jadkauskaite L, Szabó IL (2017). Oxidative damage control in a human (mini-) organ: Nrf2 activation protects against oxidative stress-induced hair growth inhibition. J Invest Dermatol.

[22] Driskell RR, Clavel C, Rendl M, Watt FM (2011). Hair follicle dermal papilla cells at a glance. J Cell Sci.

[23] Thom E (2016). Stress and the hair growth cycle: cortisol-induced hair growth disruption. J Drugs Dermatol.

[24] Xiang L, Sunesara I, Rehm KE, Marshall GD Jr (2016). Hair cortisol concentrations are associated with hair growth rate. Neuroimmunomodulation.

[25] Wang L, Guo LL, Wang LH (2015). Oxidative stress and substance P mediate psychological stress-induced autophagy and delay of hair growth in mice. Arch Dermatol Res.

[26] Azziz R, Carmina E, Sawaya ME (2000). Idiopathic hirsutism. Endocr Rev.

[27] Sharma NL, Mahajan VK, Jindal R, Gupta M, Lath A (2008). Hirsutism: clinico-investigative profile of 50 Indian patients. Indian J Dermatol.

[28] Escobar-Morreale HF, Carmina E, Dewailly D (2012). Epidemiology, diagnosis and management of hirsutism: a consensus statement by the Androgen Excess and Polycystic Ovary Syndrome Society. Hum Reprod Update.

[29] Li R, Qiao J, Yang D, Li S, Lu S, Wu X, Wei Z (2012). Epidemiology of hirsutism among women of reproductive age in the community: a simplified scoring system. Eur J Obstet Gynecol Reprod Biol.

[30] Kopera D, Wehr E, Obermayer-Pietsch B (2010). Endocrinology of hirsutism. Int J Trichology.

[31] Unluhizarci K, Kaltsas G, Kelestimur F (2012). Non polycystic ovary syndrome-related endocrine disorders associated with hirsutism. Eur J Clin Invest.

[32] Carmina E, Azziz R, Bergfeld W (2019). Female pattern hair loss and androgen excess: a report from the Multidisciplinary Androgen Excess and PCOS Committee. J Clin Endocrinol Metab.

[33] Shibli-Rahhal A, Van Beek M, Schlechte JA (2006). Cushing's syndrome. Clin Dermatol.

[34] Ellis JA, Sinclair R, Harrap SB (2002). Androgenetic alopecia: pathogenesis and potential for therapy. Expert Rev Mol Med.

[35] Sanke S, Chander R, Jain A, Garg T, Yadav P (2016). A comparison of the hormonal profile of early androgenetic alopecia in men with the phenotypic equivalent of polycystic ovarian syndrome in women. JAMA Dermatol.

[36] Singal A, Sonthalia S, Verma P (2013). Female pattern hair loss. Indian J Dermatol Venereol Leprol.

[37] Wang TL, Zhou C, Shen YW (2010). Prevalence of androgenetic alopecia in China: a community-based study in six cities. Br J Dermatol.

[38] Harrison S, Sinclair R (2002). Telogen effluvium. Clin Exp Dermatol.

